# Compounds from the Medicines for Malaria Venture Box Inhibit In Vitro Growth of *Babesia divergens*, a Blood-Borne Parasite of Veterinary and Zoonotic Importance

**DOI:** 10.3390/molecules26237118

**Published:** 2021-11-24

**Authors:** Mohamed Abdo Rizk, Shimaa Abd El-Salam El-Sayed, Mahmoud S. Alkhoudary, Khalaf F. Alsharif, Mohamed M. Abdel-Daim, Ikuo Igarashi

**Affiliations:** 1 National Research Center for Protozoan Diseases, Obihiro University of Agriculture and Veterinary Medicine, Inada-Cho, Obihiro 080-8555, Hokkaido, Japan; Shimaa_a@mans.edu.eg; 2 Department of Internal Medicine and Infectious Diseases, Faculty of Veterinary Medicine, Mansoura University, Mansoura 35516, Dakahlia, Egypt; 3 Department of Biochemistry and Chemistry of Nutrition, Faculty of Veterinary Medicine, Mansoura University, Mansoura 35516, Dakahlia, Egypt; 4 Faculty of Pharmacy, Mansoura University, Mansoura 35516, Dakahlia, Egypt; sabryelkhodery@gmail.com; 5 Department of Clinical Laboratory Sciences, College of Applied Medical Sciences, Taif University, P.O. Box 11099, Taif 21944, Saudi Arabia; alsharif@tu.edu.sa; 6 Pharmacology Department, Faculty of Veterinary Medicine, Suez Canal University, Ismailia 41522, Egypt; abdeldaim.m@vet.suez.edu.eg

**Keywords:** *Babesia divergens*, malaria box, in vitro, large-scale screening, MMV006913

## Abstract

Babesiosis is an infectious disease with an empty drug pipeline. A search inside chemical libraries for novel potent antibabesial candidates may help fill such an empty drug pipeline. A total of 400 compounds (200 drug-like and 200 probe-like) from the Malaria Box were evaluated in the current study against the in vitro growth of *Babesia divergens* (*B. divergens*), a parasite of veterinary and zoonotic importance. Novel and more effective anti-*B. divergens* drugs than the traditionally used ones were identified. Seven compounds (four drug-like and three probe-like) revealed a highly inhibitory effect against the in vitro growth of *B. divergens*, with IC_50_s ≤ 10 nanomolar. Among these hits, MMV006913 exhibited an IC_50_ value of 1 nM IC_50_ and the highest selectivity index of 32,000. The atom pair fingerprint (APfp) analysis revealed that MMV006913 and MMV019124 showed maximum structural similarity (MSS) with atovaquone and diminazene aceturate (DA), and with DA and imidocarb dipropionate (ID), respectively. MMV665807 and MMV665850 showed MMS with each other and with ID. Of note, a high concentration (0.75 IC_50_) of MMV006913 caused additive inhibition of *B. divergens* growth when combined with DA at 0.75 or 0.50 IC_50_. The Medicines for Malaria Venture box is a treasure trove of anti-*B. divergens* candidates according to the obtained results.

## 1. Introduction

Babesiosis is a tick-borne parasitic disease causing serious economic losses in the livestock industry worldwide [1]. In general, babesiosis is manifested by fever, hemoglobinuria, hemolytic anemia, jaundice, and death [2,3].

*Babesia divergens* (*B. divergens*) is considered one of the main causes of veterinary and human babesiosis in Europe [3]. The currently available drugs used to control *Babesia* infection in animals, imidocarb dipropionate (ID) and diminazene aceturate (DA), have proven their limitations regarding toxicity to hosts and parasite resistance [4]. Safety and residue problems have led to the withdrawal of these anti-*B. divergens* drugs from the market [5]. Severe cases of human babesiosis have not responded to treatment with the most commonly used anti-*B. divergens* drugs, such as clindamycin, azithromycin, quinine, atovaquone, and tetracycline [5]. Therefore, the discovery and development of more effective and safer antibabesial agents have become an urgent need. The Malaria Box, a collection of 400 compounds, was designed to be the starting point for drug discovery for *Plasmodium falciparum* (*P. falciparum*) and other medically important pathogens [6]. Since *Plasmodium* and *Babesia* parasites belong to the phylum Apicomplexa and both of them have close biological similarities, in the present study, we have evaluated the inhibitory effects of Malaria Box compounds against the in vitro growth of a *Babesia* species with veterinary and zoonotic importance, namely *B. divergens*.

The in vitro potency of a given chemotherapeutic can be a good preclinical marker of the therapeutic potential in vivo [7]. In this regard, promising MMV compounds for further development as novel therapies for animal or human babesiosis were identified through this study, reflecting the importance of the Malaria Box as a rich source of potential screening hits and biological probes for non-*Plasmodium* applications, which might help fill the empty anti-*Babesia* drug pipeline.

## 2. Results and Discussion

The Malaria Box is a valuable source of commercially available compounds, representing families of structures identified in phenotypic screens of pharmaceutical and academic libraries against *P. falciparum*, and was made available to identify novel drugs to combat other apicomplexan parasites [8]. Malaria Box, a collection of 400 compounds, is divided into 200 diverse drug-like compounds and 200 diverse probe-like ones [6]. The compounds that are absorbed by the oral route and toxophores are defined as drug-like compounds. Other hits are assigned to the probe-like category [2,8]. The in vitro inhibitory effects of Malaria Box compounds against 16 protozoa, 7 helminths, 9 bacterial and mycobacterial species, and the NCI60 human cancer cell line were published within a large, complete dataset [6]. The promising candidates identified from such wide screening of Malaria Box compounds, together with the zoonotic importance of *B. divergens*, encouraged us to perform, in the present study, a large-scale in vitro screening of MMV compounds (*n* = 400) against *B. divergens*. 

In fact, a previous study [9] measured the susceptibilities of multiple blood-stage *Plasmodium* and *Babesia* parasite species to the Malaria Box compounds to investigate the possible sharing of the erythrocyte-specific apicomplexans to chemical sensitivities during the clinically relevant stages of parasitic infection. In this previous study, the authors evaluated the inhibitory effects of MMV compounds from Malaria Box against *B. divergens* only without either determination of the parasite viability after stopping the treatment, evaluating the possible synergistic or antagonistic interaction between MMV compounds and other antibabesial drugs, or preforming any bioinformatic analysis to the identified potent MMV hits. In our study, we have evaluated the inhibitory effects of MMV compounds from the Malaria Box on *B. divergens* growth. Then, we have evaluated the parasite viability after four days of treatments by the identified potent MMV hits. Of note, we have calculated the structural similarity between the drug-like MMV compounds and drugs commonly used to treat babesiosis using APfp. Additionally, combination therapies of MMV compounds with the lowest IC_50_s and the highest SIs with DA against the in vitro growth of *B. divergens* parasite were evaluated in our study. In the study performed by Paul et al. [9], the authors evaluated the inhibitory effects of MMV compounds from Malaria Box against *B. divergens* using a [^3^*H*]-hypoxanthine uptake assay. Indeed, radiolabeled precursors of phospholipids, such as the sources of phospholipid polar head groups, [^3^*H*]-hypoxanthine, are widely used radioisotopes for the antimalarial in vitro drug-sensitivity assay [10,11,12]. This assay is based on the inability of either platelets or uninfected erythrocytes to synthesize their RNA, DNA, and proteins, or their membranes or leukocytes to multiply during the in vitro malaria parasite culture. Thus, only malaria parasites have actively dividing cells during the in vitro culture process, and the addition of radioactive substances into the culture enables the parasites to integrate the radioactive precursors themselves, which is considered an indirect measurement of the parasite metabolic activity [10,11]. Generally, *Plasmodium* spp. are unable to synthesize purines de novo [13], which force *Plasmodium* parasites to use exogenous purines. Therefore, the incorporation of a radioisotope, such as [^3^*H*]-hypoxanthine, which is the main purine base needed by *P. falciparum*, is directly related to the count of *P. falciparum*-infected erythrocytes [11,12]. For *Babesia* parasites, there is paucity on the occurrence or the absence of de novo synthetic pathways of purines. Studies on the uptake of purines by *Babesia*-infected erythrocytes indicate that hypoxanthine is likely to be the major purine source in vivo [14]. For *B. divergens*, the findings indicate that the parasite can synthesize purine nucleotides by two routes, one involving PRTases and the other employing nucleoside kinases [15]. Thus, the parasite does not need to use exogenous purines. Subsequently, the incorporation of a radioisotope, such as [^3^*H*]-hypoxanthine, with the parasite does not have a significant value for counting of *Babesia*-infected erythrocytes. Therefore, in the current study, we used a recently established, accurate, and automatic fluorescence assay using SGI stain to screen the huge library-like Malaria Box against the in vitro growth of *B. divergens*.

The in vitro inhibitory effects of MMV Malaria Box compounds against the growth of the *B. divergens* parasite were varied: potent (IC_50_ < 1µM, *n* = 156), moderate (IC_50_ 1–10 µM, *n* = 186), or weak (IC_50_ > 10µM, *n* = 58) (Tables S1–S20). Subsequently, Malaria Box compounds exhibited a 39% hit rate against the in vitro growth of the screened parasite (Figure S1). This hit rate is higher than those caused by Malaria Box compounds against the in vitro growth of *Babesia bovis*, *Babesia bigemina*, *Theileria equi*, and *Babesia caballi*, respectively (Figure S1) [2]. Such high in vitro susceptibility of *B. divergens* to compounds derived from Malaria Box may be attributed to the fact that *B. divergens* and *P. falciparum* use common receptors, glycophorins A and B, to invade the infected red blood cell [16]. Future studies are required to explore whether glycophorin receptors located on the RBCs’ surface are drug targets where the identified potent MMV compounds produce their inhibitory effects in *B. divergens* infection. Indeed, previous studies [17,18,19,20,21] have demonstrated that certain compounds other than MMV compounds could inhibit the parasite invasion of RBCs through targeting the parasite secretory proteins on RBCs in both *B. divergens* and *P. falciparum*. Boyle, et al. [19] reported that heparin inhibits the invasion process of *P. falciparum* through inhibiting the earliest step in the invasion of merozoite to RBCs, initial RBC attachment, and binding merozoite surface protein 1 (MSP1). Additionally, heparin inhibits the merozoite invasion of RBCs in *P. falciparum* by binding to rhoptry and microneme proteins that are involved in reorientation and signaling steps of the invasion process [18,20]. For *Babesia* spp. (*B. bovis, B. bigemina, B. equi*, and *B. caballi* in vitro and *B. microti* in vivo), Bork, et al. [17] established that heparin covers the surfaces of babesial merozoites and inhibits their subsequent invasion of erythrocytes.

The hit rate of MMV compounds against the in vitro growth of *B. divergens* is considered higher than that observed by the in vitro screening of Malaria Box compounds against either *Toxoplasma gondii* (*T. gondii*) and *Entamoeba histolytica* [22], *Schistosoma mansoni* [23], or *Cryptosporidium parvum* [24] (Figure S1). 

**Figure 1:**
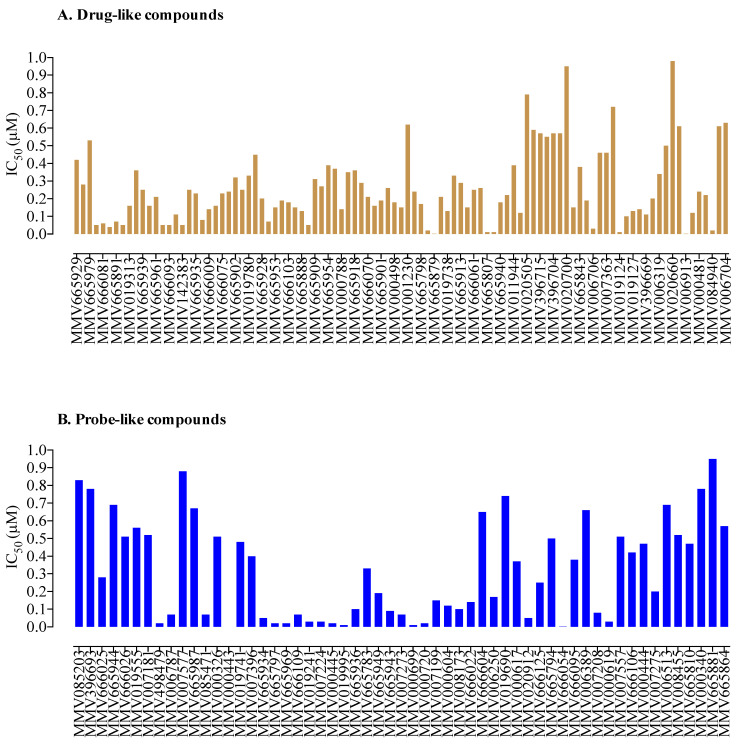
Drug-like and probe-like compounds with potential (IC_50_ < 1 µM) against *B. divergens* identified from in vitro screening of the Malaria Box. (**A**). Drug-like compounds. (**B**). Probe-like compounds. The data are the means of two independent experiments.

**Figure 2:**
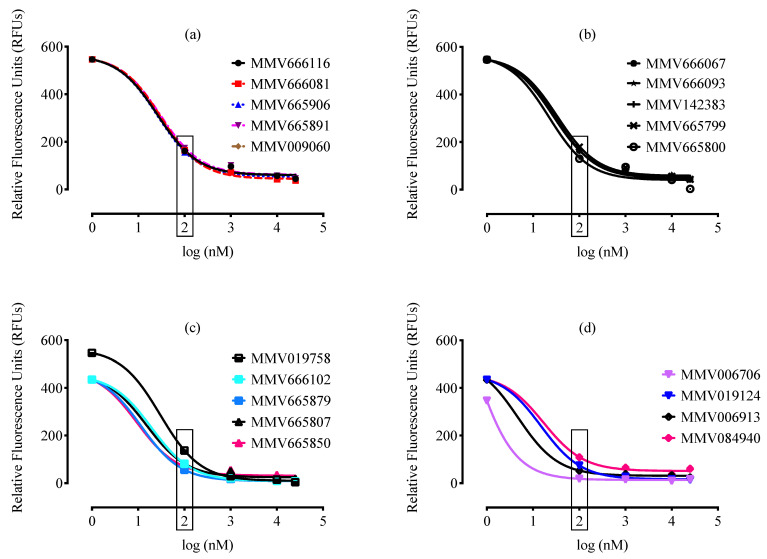
Inhibition of *B. divergens* in vitro growth by MMV drug-like compounds. (**a**). Relationship between MMV666116, MMV666081, MMV665906, MMV665891, and MMV009060 concentrations (nM) and RFUs for the parasite. (**b**). Relationship between MMV666067, MMV666093, MMV142383, MMV665799, and MMV665800 concentrations (nM) and RFUs for the parasite. (**c**). Relationship between MMV019758, MMV666102, MMV665879, MMV665807, and MMV665850 concentrations (nM) and RFUs for the parasite. (**d**). Relationship between MMV006706, MMV019124, MMV006913, and MMV084940 concentrations (nM) and RFUs for the parasite. Each value is presented as the mean of three independent experiments after subtraction of the background fluorescence for nonparasitized RBCs. The lowest drug concentrations for which inhibition of *B. divergens* growth was statistically significant (*p* < 0.05) are indicated by a rectangle.

**Figure 3:**
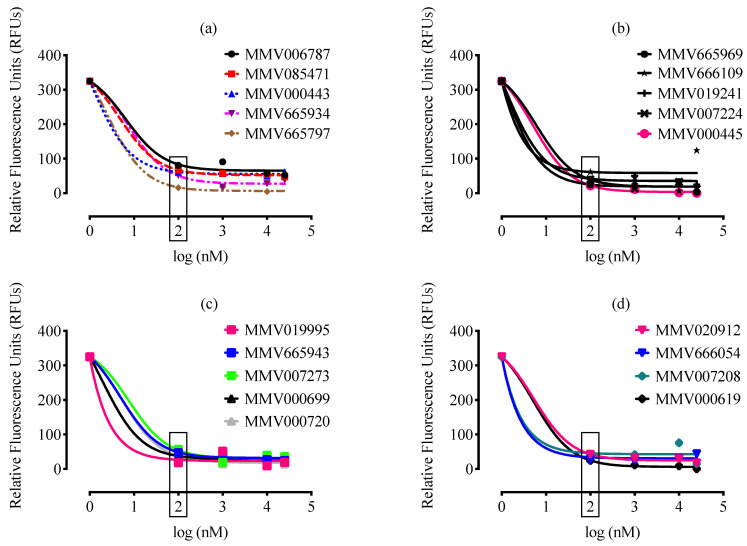
Inhibition of *B. divergens* in vitro growth by MMV probe-like compounds. (**a**). Relationship between MMV006787, MMV085471, MMV000443, MMV665934, and MMV665797 concentrations (nM) and RFUs for the parasite. (**b**). Relationship between MMV665969, MMV666109, MMV019241, MMV007224, and MMV000445 concentrations (nM) and RFUs for the parasite. (**c**). Relationship between MMV019995, MMV665943, MMV007273, MMV000699, and MMV000720 concentrations (nM) and RFUs for the parasite. (**d**). Relationship between MMV020912, MMV666054, MMV007208, and MMV000619 concentrations (nM) and RFUs for the parasite. Each value is presented as the mean of three independent experiments after subtraction of the background fluorescence for nonparasitized RBCs. The lowest drug concentrations for which inhibition of *B. divergens* growth was statistically significant (*p* < 0.05) are indicated by a rectangle.

A total of 99 MMV drug-like and 57 MMV probe-like compounds exhibited potent anti-*B. divergens* activity in vitro, with nanomole levels of IC_50_ (Figure 1). From these compounds, 38 compounds (19 drug-like and 19 probe-like) were more effective than DA (Figure 2 and Figure 3, Figure S2, and Tables S1–S20). Treatment with 100 nM of these potent 38 compounds significantly inhibited (*p* < 0.05) the in vitro growth of *B. divergens* (Figure 2 and Figure 3). 

**Figure 4:**
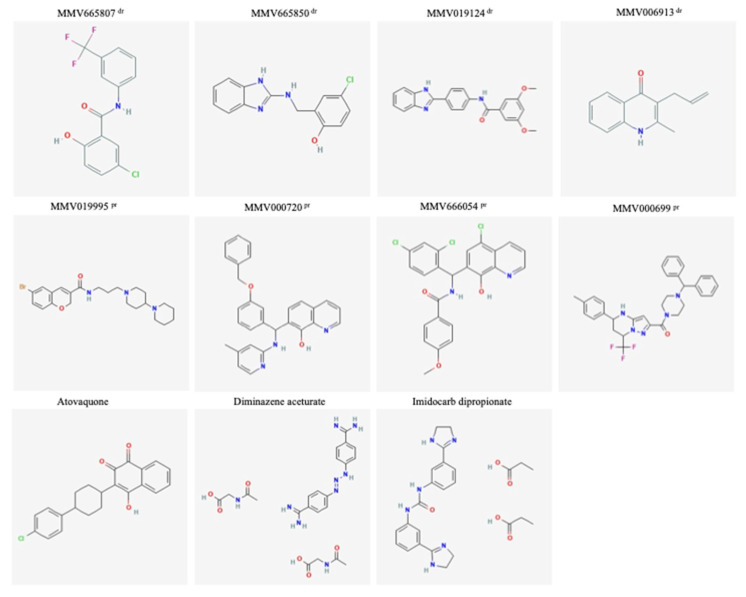
Chemical structures of drug-like and probe-like compounds exhibited a high potential (IC_50_ ≤ 10 nM) against *B. divergens* identified from in vitro screening of the Malaria Box. MMV structures were provided by the MMV as part of the supporting information for the Malaria Box. ^dr^ drug-like compounds. ^pr^ probe-like compound.

Interestingly, seven compounds (four drug-like and three probe-like) exhibited a highly inhibitory effect against the in vitro growth of *B. divergens*, with IC_50_s less than 10 nanomolar (Table 1 and Figure 4). MMV019124, MMV006913, MMV019995, MMV000699, and MMV666054 exhibited IC_50_s lower than those observed for *P. falciparum* (Table 1). In general, several characteristics associated with the screening parasite, such as parasite species, strain, and size, have an impact on the therapeutic efficacy of the tested drugs [2,4]. In vitro culture parameters, such as the utilized medium, HCT, and the presence or absence of serum, also influence the calculated IC_50_s of the tested medication [25,26]. As a result, differences in parasite species or culture conditions between *Babesia* and *Plasmodium* might explain the inconsistencies in MMV compound IC_50_ values between two parasites.

Of note, MMV006913 was the most promising hit for further medicinal chemistry/biological screenings, with SI 32000 and IC_50_ 1 nM (Table 1). To the best of our knowledge, the inhibitory effect of this hit in an animal model of babesiosis remains unknown. Moreover, the mechanism by which MMV006913 inhibits either *Plasmodium* or *Babesia* needs more deep investigation. Some theories suggested cGMP-dependent or mitogen-activated protein kinases (PK) as targets of this MMV hit in the *Plasmodium* parasite [6]. Generally speaking, PK has important physiological functions in the mammalian system, e.g., in vascular and gastrointestinal smooth muscles, platelets, kidneys, bones, and the central nervous system [27]. Among protozoan parasites, PKs are found in *P. falciparum*, *T. gondii*, and *Babesia* spp. [27]. The PK similarity between *Babesia* and *P. falciparum* is over 70% [27], possibly explaining why antimalarial compounds that target the PK gene are effective against *Babesia* parasites.

The inhibitory assay results obtained in the current study showed some differences from those previously published by Paul et al. [9]. Several factors affect the parasite susceptibility to the screened chemicals, including parasite strain and species, the inhibitory assay used for chemical screening, and the in vitro culture conditions [4,26]. Subsequently, the differences in the used parasite strain (Rouen 1987 vs. German bovine strain), the used inhibitory assay (^3^*H*-hypoxanthine uptake assay vs. fluorescence assay using SGI stain assay), and the in vitro culture conditions (standard hypoxic conditions (e.g., 1% O_2_, 5% CO_2_, 94% N2), 2%-hematocrit, RPMI 1640 medium supplemented with 25 mM HEPES, 50 mg/l hypoxanthine, 2.42 mM sodium bicarbonate, and 4.31 mg/mL AlbuMAX II, 6.75 pH of the media vs. RPMI 1640 medium containing 40% normal bovine serum; 60 units per milliliter of penicillin G, 60 µg/mL of streptomycin, and 0.15 µg/mL of amphotericin B; cultures of parasitized RBCs (pRBCs) incubated at 37 °C in an atmosphere of 5% CO_2_, 5% O_2_, and 90% N_2_), explain the discrepancies in the results between our results and the results obtained from the previous study that screened Malaria Box compounds against *B. divergens* parasite [9].

To determine the ability of potent identified MMV hits to suppress the regrowth of *B. divergens* after four days of in vitro treatment, viability testing was performed. Results showed that there was no regrowth of the screened parasite after treatment by all MMV compounds, even at the lowest concentration (0.1 µM) (Table S21). 

**Figure 5:**
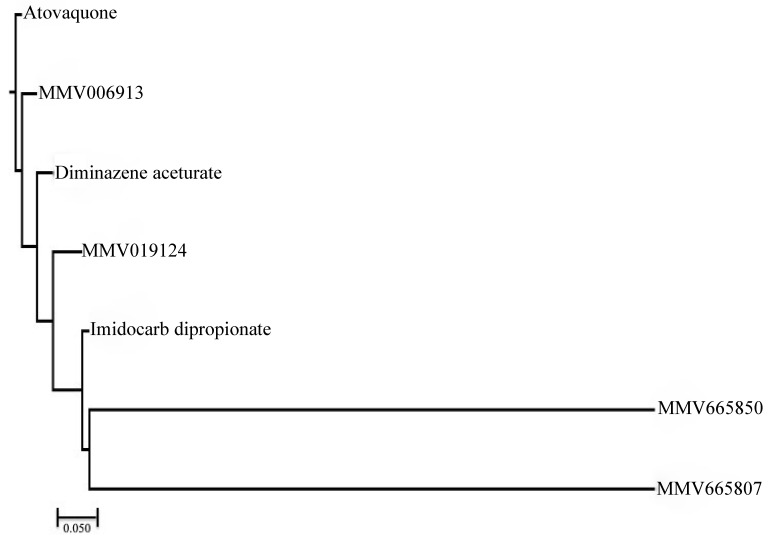
Hierarchical clustering analysis. The hierarchical clustering analysis was performed using ChemmineR software and highlights the structural similarities between the MMV compounds with potent anti-*B. divergens* activity and the commonly used antibabesial drugs (diminazene aceturate, imidocarb dipropionate, and atovaquone). Dissimilarity is reported using a scale unit of 0.05.

The HCA analysis revealed that MMV006913 (CID: 780973) showed the maximum structural similarity with either atovaquone or DA (Figure 5). In the same way, the MMV019124 compound (CID: 1347726) exhibited the maximum structural similarity with either DA or ID (Figure 5). MMV665807 (CID: 5065884) and MMV665850 (787413) showed the maximum structural similarity with each other or with ID (Figure 5). Compounds falling under different clusters indicate structural dissimilarity among them and in comparison with other commonly used antibabesial drugs, indicating a probable different antibabesial mechanism. Subsequently, MMV hits that showed the lowest IC_50_s against the growth of *B. divergens* and the highest SIs (MMV006913 and MMV666054) were selected to further evaluate their inhibitory effects when used in combination with the commonly used antibabesial drug, DA. All combination ratios of MMV006913, when used with DA, revealed additive and indifference inhibitory effects against the in vitro growth *B. divergens* (Table 2). Of note, a very high concentration (0.75 IC_50_) of MMV006913 caused an additive interaction on the growth of *B. divergens* when used in combination, with either 0.75 IC_50_ or 0.50 IC_50_ of DA (Table 2). On the contrary, the combined use of MMV666054 and DA revealed indifference and antagonistic interactions regarding the in vitro growth of *B. divergens* (Table 2). Further in vivo experiments are required to confirm the in vitro enhancement in the inhibitory effect of MMV006913 when used in combination with DA. 

Of the hits with an IC_50_ less than 10 nM, five compounds (two drug-like and three probe-like) exhibited SI ranging from 1195.64 to 7785.47 (Table 1 and Figure 4). Future in vivo experiments are required to evaluate the inhibitory effects of these MMV compounds using *B. microti* as the infectious agent.

## 3. Materials and Methods

### 3.1. Chemical Reagents

SYBR Green I (SGI) nucleic acid stain (Lonza, Rockland, NY, USA; 10,000×) was stored at –20 °C and thawed before use. A lysis buffer consisting of Tris (130 mM; pH 7.5), ethylenediaminetetraacetic acid (EDTA) (10 mM), saponin (0.016%; *w/v*), and TritonX-100 (1.6%; *v*/*v*) was prepared in advance and stored at 4 °C. DA (Novartis, Tokyo, Japan) and MMV compounds (MolPort, Riga, Latvia) were prepared as a 100 mM stock solution and stored at –30 °C until use. The Malaria Box compounds were supplied in V-shaped 96-well plates in 20 µL of a 10 mM DMSO (Sigma-Aldrich, Tokyo, Japan) solution and shipped frozen. Plate mapping and full data on the Malaria Box with original GSK/St. Jude/Novartis compound number, ID, set, molecular weight, canonical SMILES, source, and biological data are provided as supporting information for the Malaria Box (in pone.0062906.s002-supporting information). A list of vendors used to supply compounds for the Malaria Box, including the vendor’s web address and the number of compounds from each vendor in the Malaria Box, is also available (in pone.0062906.s002-supporting information).

### 3.2. In Vitro Cultivation of B. divergens Parasite

*Babesia divergens* (German bovine strain of catalog number 201708-4) used in this study were cultivated in purified bovine red blood cells (RBCs) using a microaerophilic, stationary-phase culture system [26,28]. Briefly, *B. divergens* [26,29] was cultivated in RPMI 1640 medium (Sigma-Aldrich) containing 40% normal bovine serum. Sixty units per milliliter of penicillin G, 60 µg/mL of streptomycin, and 0.15 µg/mL of amphotericin B (all from Sigma-Aldrich) were added to all of the culture medium used. Antibiotics were added to the parasite culture at a concentration that did not harm the RBCs to prevent bacterial contamination and, subsequently, minimize the loss of valuable cells, reagents, time, and effort due to contamination. Cultures of parasitized RBCs (pRBCs) were incubated at 37 °C in an atmosphere of 5% CO_2_, 5% O_2_, and 90% N_2_.

### 3.3. Malaria Box Compound Screening against B. divergens 

The inhibitory effects of 400 compounds (200 drug-like and 200 probe-like) from the Malaria Box on *B. divergens* growth were tested using the fluorescence-based high-throughput screening (HTS) assay [26]. Briefly, *B. divergens* pRBCs were cultivated in 96-well plates at 1% parasitemia with nonparasitized RBCs to 5% hematocrit (HCT). RPMI 1640 medium alone or with indicated concentrations of 400 MMV compounds (0.1, 1, 10, and 25 µM) in 100 µL final volume was added to the culture. The medium containing 0.1% dimethyl sulfoxide (DMSO) with infected RBCs (iRBCs) and uninfected RBCs was used as positive and negative controls, respectively. DA was used as the positive control and tested at a final concentration of 10 µM. One plate was used in each drug experiment for 50% inhibitory concentration (IC_50_) value calculation on the fourth day of culture. A lysis buffer containing SGI was added to each well, as reported above. The mean fluorescence values were then plotted against the logarithm of drug concentrations. Selectivity indices (SIs) for the identified potent compounds were calculated as the ratio of the cytotoxic concentration 50 (CC_50_) to the IC_50_ for each MMV compound. The cytotoxicity was evaluated using the human fibroblast cell line MRC-5 and retrieved from ChEMBL.

### 3.4. Viability Test 

MMV hits that exhibited potent anti-*B. divergens* efficacy with IC_50_ ≤ 10 nM were selected to evaluate the *B. divergens* parasite viability after four days of treatments by these compounds, as previously described by [26]. Briefly, experiments similar to those described in Section 3.3 were performed. On the fourth day of treatment, 3.5 µL of the DMSO- or MMV-treated infected RBCs were removed and diluted in a fresh growth medium. Then, the plates were incubated at 37 °C for the next four days. The amount of parasite DNA was measured using a fluorescence spectrophotometer and used as an indicator of parasite recrudescence. Each experiment was repeated three times. 

### 3.5. Structural Similarity Measurements

To calculate the structural similarity between the drug-like MMV compounds (*n* = 4) and drugs commonly used to treat babesiosis (DA, ID, and atovaquone), we used atom pair fingerprints (APfp) [30]. APfp was calculated using the chemical ID obtained from the PubChem for each compound. The CIDs were then loaded into the ChemMine tools software for calculating APfp of all compounds [31]. The APfp was submitted to ChemmineR software for hierarchical clustering analysis (HCA) [32].

### 3.6. In Vitro Drug Combination Test

Combination therapies of MMV compounds (MMV006913 and MMV666054) that exhibited the lowest IC_50s_ (1 nM) and the highest SIs with the commonly used antibabesial drug DA against the in vitro growth of *B. divergens* parasite were evaluated. The combination ratios ranged from 0.75 IC_50_ MMV compounds:0.75 IC_50_ DA to 0.50 IC_50_ MMV compounds:0.50 IC_50_ DA (Table 2), as previously described [3], with some modifications. Each combination was loaded in triplicate wells in 96-well plates and incubated with the parasite at 1% parasitemia and 5% HCT for 4 consecutive days. RBCs collected from healthy cattle were loaded into each well in triplicate with the medium and DMSO and used as blank controls. On the fourth day of culture, 100 µL of a lysis buffer containing a 2 × SGI nucleic acid stain was added directly to each well on the plates, and then the plates were incubated for 6 h in a dark place at room temperature and the fluorescence values were determined as described above. Three independent experiments were performed for each combination therapy.

### 3.7. Statistical Analyses

The IC_50_ values of MMV compounds were calculated using the nonlinear regression curve fit in GraphPad Prism 5.0 (GraphPad Software, Inc., San Diego, CA, USA). The differences in the emitted fluorescence signals for the in vitro cultures and drug combination test were analyzed with a commercial statistical software program (GraphPad Prism) using the independent unpaired Student’s *t*-test [33]. A *p* value of < 0.05 was considered statistically significant for all tests. The regrowth of the parasite in the viability test was determined based on the statistically significant differences between the MMV-hits-treated and the positive control group (*Babesia* treated with RPMI 1640 medium containing the vehicle only, DMSO) [26].

## 4. Conclusions

Screening the Malaria Box compounds against the in vitro growth of *B. divergens* assisted in the discovery of new, more effective drugs than the traditionally used ones, either at the animal level, DA and ID, or human level, clindamycin, azithromycin, and quinine. Attractively, seven novel potent anti-*B. divergens*, including MMV665807, MMV665850, MMV019124, MMV006913, MMV019995, MMV000699, and MMV666054, were identified. One hit, MMV006913, exhibited the lowest IC_50_ and the highest selectivity indices, highlighting the possible promising in vivo inhibitory effect of this hit when administrated in either a mouse infected with *B. microti* or gerbil infected by *B. divergens*. Seven other MMV hits showed excellent SIs. Although the present study helped in the discovery of novel MMV with potential anti-*B. divergens* efficacy, the inhibitory effects of these hits may be enhanced when used in combination with each other. Future studies are required to analyze the two-drug interactions of MMV hits when used in combination with each other for further clinical application. The obtained results indicate that the Malaria Box is a treasure trove of anti-*B. divergens* candidates. Knowledge of the targets and action mechanisms of these compounds might provide new insights into *Babesia* biology. Therefore, further researches are essential to calculate the distance matrix between the identified MMV compounds with anti-*B. divergens* activity and the commonly used antibabesial drugs, including highlighting the structural differences between these compounds using PubChem fingerprint for similarity workbench. Furthermore, future studies are required to establish a simple mathematical relationship between structural or property structural molecular features (descriptors) of the identified potent MMV compounds and their physicochemical activities using quantitative structure–activity relationships (QSAR). Such physicochemical descriptors, which include parameters to account for hydrophobicity, topology, electronic properties, and steric effects, will be determined empirically or, more recently, by computational methods. Three main steps are required for accomplishing QSAR modeling: (1) collect or, if possible, design a training set of chemicals; (2) choose descriptors that can properly relate chemical structure to biological activity; and (3) apply statistical methods that correlate changes in structure with changes in biological activity.

## Figures and Tables

**Table 1:** MMV hits with potent inhibitory activity against *B. divergens* in vitro.

Compound ID ^a^	Set *	MW * (g/mol)	IC_50_ (nM) * P. falciparum	IC_50_ (nM) ^b^ *B. divergens*	CC_50_ (nM) ^c^ Against MRC-5	SI ^d^
MMV665807	Drug-like	315.67	ND	10	240	24
MMV665850	Drug-like	273.71	ND	10	15878	1587
MMV019124	Drug-like	373.40	1089.5	10	32000	3200
MMV006913	Drug-like	199.24	1290	1	32000	32000
MMV019995	Probe-like	462.42	563	10	11956	1195
MMV000699	Probe-like	559.62	454	10	32000	3200
MMV666054	Probe-like	487.76	513	1	7785	7785

^a^ Compounds are designated by their MMV identifier codes. ^b^ IC_50_ values for each drug were calculated on the fourth day based on the growth inhibitions determined using the fluorescence-based method. Results are means from duplicate experiments. ^c^ Cytotoxicity data were evaluated using the human fibroblast cell line MRC-5 and retrieved from the CHEMBL database (https://www.ebi.ac.uk/chembl/, accessed on August 2021). ^d^ Selectivity indices (SIs) are the ratios of CC_50_ values over IC_50_ values for the MMV compounds tested. ND, not determined. * The set, molecular weight (MW), and *P. falciparum* IC_50_ data are provided in the supporting information for the open-access Malaria Box.

**Table 2:** Effects of diminazene aceturate combined with MMV006913 or MMV666054 on in vitro growth of *B. divergens*.

Drug Combination	C ^a^	FIC_D1_	FIC_D2_	ΣFIC	Degree of Interaction ^b^
MMV006913 + DA	0.75 + 0.75	0.41	0.46	0.87	Additive
	0.75 + 0.50	0.47	0.39	0.86	Additive
	0.50 + 0.75	0.74	0.93	1.67	Indifference
	0.50 + 0.50	0.88	1.03	1.91	Indifference
MMV666054 + DA	0.75 + 0.75	1.6	0.22	1.82	Indifference
	0.75 + 0.50	1.01	0.1	1.11	Indifference
	0.50 + 0.75	1.29	0.05	1.34	Indifference
	0.50 + 0.50	2.51	0.51	3.02	Antagonism

^a^ C refers to the different concentrations of potent MMV hits with IC_50s_ = 1 nM against *B. divergens* in combination with diminazene aceturate. ^b^ The degree of drug interaction was determined based on the following fractional inhibitory concentration (FIC) index: >0.5–1 (additive), >1 to <2 (indifferent), and ≥2 (antagonistic). FIC_D1_ refers to the fractional inhibitory concentration of either MMV006913 or MMV666054. FIC_D2_ refers to the fractional inhibitory concentration of diminazene aceturate (DA).

## Data Availability

The datasets generated during and/or analysed during the current study are available from the corresponding author on reasonable request.

## Supplementary Materials

The following are available online, Figure S1. A heatmap showing the hit rates percentages for the Malaria Box compounds against several piroplasm parasites (B. divergens, B. bovis, B. bigemina, T. equi, and B. caballi), Toxoplasma gondii, Entamoeba histolytica, Schistosoma mansoni, and Cryptosporidium parvum. B. divergens exhibited the highest hit rate %.* data adopted from [2], # data adopted from [22], ** data adopted from [23], ## data adopted from [24]. Figure S2. Fluorescence-based monitoring of diminazene aceturate-induced growth inhibition of B. divergens. Correlation between RFUs (y-axis) and log concentration of diminazene aceturate (nM) (x-axis). Each value represents a mean of triplicate wells after subtraction of the background fluorescence for non-parasitized RBCs. Table S1: Characterization and IC50 values of malaria box compounds number 2 to 21 from plate (A) evaluated for 2; Table S2: Characterization and IC50 values of malaria box compounds number 22 to 41 from plate (A) evaluated for 8; Table S3: Characterization and IC50 values of malaria box compounds number 42 to 61 from plate (A) evaluated for 23; Table S4: Characterization and IC50 values of malaria box compounds number 62 to 81 from plate (A) evaluated for Babesia divergens parasites; Table S5: Characterization and IC50 values of malaria box compounds number 82 to 101 from plate (B) evaluated for Babesia divergens parasites; Table S6: Characterization and IC50 values of malaria box compounds number 102 to 121 from plate (B) evaluated for Babesia divergens parasites; Table S7: Characterization and IC50 values of malaria box compounds number 122 to 141 from plate (B) evaluated for Babesia divergens parasites; Table S8. Characterization and IC50 values of malaria box compounds number 142 to 161 from plate (B) evaluated for Babesia divergens parasites; Table S9: Characterization and IC50 values of malaria box compounds number 162 to 181 from plate (C) evaluated for Babesia divergens parasites; Table S10: Characterization and IC50 values of malaria box compounds number 182 to 201 from plate (C) evaluated for Babesia divergens parasites; Table S11: Characterization and IC50 values of malaria box compounds number 202 to 221 from plate (C) evaluated for Babesia divergens parasites; Table S12: Characterization and IC50 values of malaria box compounds from 222 to 241 from plate (C) evaluated for Babesia divergens parasites; Table S13: Characterization and IC50 values of malaria box compounds number 242 to 261 from plate (D) evaluated for Babesia divergens parasites; Table S14: Characterization and IC50 values of malaria box compounds number 262 to 281 from plate (D) evaluated for Babesia divergens parasites; Table S15: Characterization and IC50 values of malaria box compounds number 282 to 301 from plate (D) evaluated for Babesia divergens parasites; Table S16: Characterization and IC50 values of malaria box compounds number 302 to 321 from plate (D) evaluated for Babesia divergens parasites; Table S17: Characterization and IC50 values of malaria box compounds number 322 to 341 from plate (E) evaluated for Babesia divergens parasites; Table S18: Characterization and IC50 values of malaria box compounds umber 342 to 361 from plate (E) evaluated for Babesia divergens parasites; Table S19: Characterization and IC50 values of malaria box compounds number 362 to 381 from plate (E) evaluated for Babesia divergens parasites; Table S20: Characterization and IC50 values of malaria box compounds number 382 to 401 from plate (E) and diminazene aceturate evaluated for Babesia divergens parasites; Table S21: Viability test results of potent MMV hits evaluated for Babesia divergens.

## Sample Availability

Samples of the compounds are available from the authors.
