# Supplementary material for: Compounds from the Medicines for Malaria Venture Box Inhibit In Vitro Growth of Babesia divergens, a Blood-Borne Parasite of Veterinary and Zoonotic Importance
Source: Molecules. 2021 Nov 24;26(23):7118. doi: 10.3390/molecules26237118 (PMC8658764; doi:10.3390/molecules26237118)
Supplement: Supplementary file 1 [file molecules-26-07118-s001.zip › molecules-1422014-supplementary.pdf]

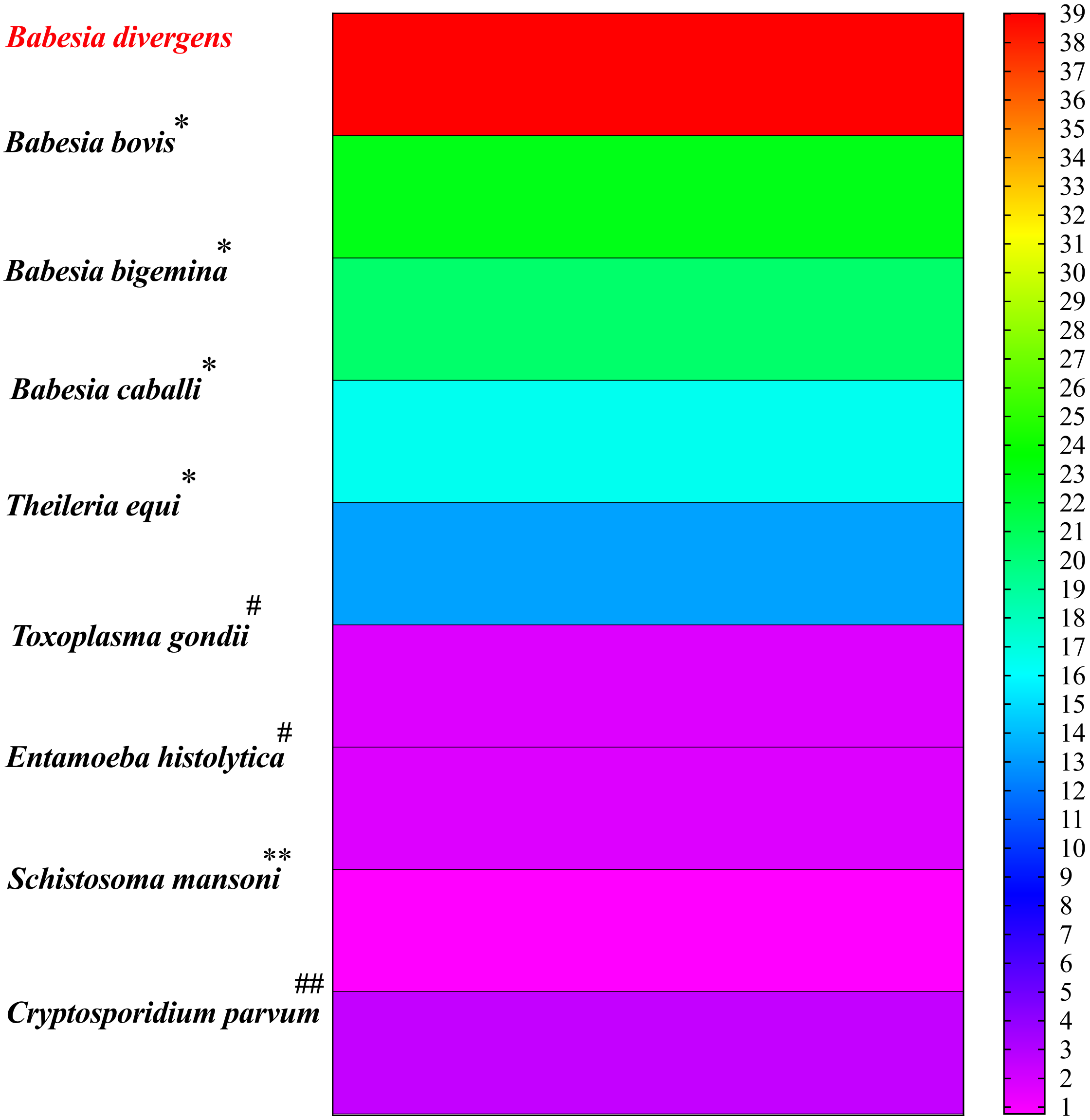

Figure S1. A heatmap showing the hit rates percentages for the Malaria Box compounds against several piroplasm parasites (*B. divergens, B. bovis*, *B. bigemina*, *T. equi*, and *B. caballi), Toxoplasma gondii*, *Entamoeba histolytica, Schistosoma mansoni,* and *Cryptosporidium parvum.* *B. divergens* exhibited the highest hit rate %.* data adopted from **[2],** # data adopted from **[22]**, ** data adopted from **[23]**, ## data adopted from **[24]**.

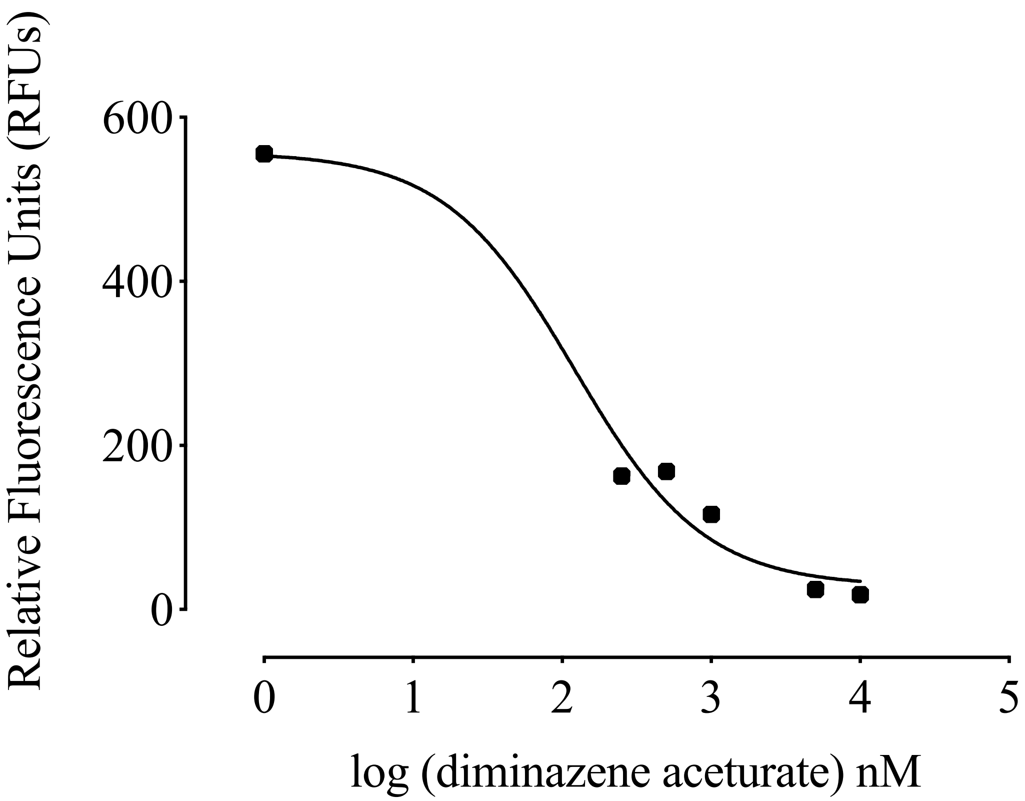

Figure S2. Fluorescence-based monitoring of diminazene aceturate-induced growth inhibition of B. divergens. Correlation between RFUs (y-axis) and log concentration of diminazene aceturate (nM) (x-axis). Each value represents a mean of triplicate wells after subtraction of the background fluorescence for non-parasitized RBCs.

Table S1. Characterization and IC_{50} values of malaria box compounds number 2 to 21 from plate (A) evaluated for *Babesia divergens* parasites

**Compound No.**	**Compound ID** [a]	**Set***	**IC_{50} (μM)** [b]
**A-2**	MMV665915	Drug-like	10.53
**A-3**	MMV666023	Probe-like	7.34
**A-4**	MMV665876	Drug-like	15.97
**A-5**	MMV019406	Probe-like	10.52
**A-6**	MMV019871	Drug-like	1.45
**A-7**	MMV666596	Probe-like	9.07
**A-8**	MMV006309	Probe-like	1.87
**A-9**	MMV000642	Probe-like	1.98
**A-10**	MMV396672	Drug-like	10.15
**A-11**	MMV001038	Drug-like	3.04

**B-2**	MMV665916	Drug-like	2.88
**B-3**	MMV666101	Probe-like	10.27
**B-4**	MMV665841	Probe-like	10.51
**B-5**	MMV007695	Probe-like	10.55
**B-6**	MMV020788	Probe-like	5.72
**B-7**	MMV009063	Drug-like	10.12
**B-8**	MMV007384	Probe-like	1.72
**B-9**	MMV006429	Drug-like	1.16
**B-10**	MMV007116	Drug-like	1.71
**B-11**	MMV396703	Drug-like	2.62

[a] Compounds are designated by their MMV identifier codes; [b] IC_{50} values for each drug were calculated on the fourth day based on the growth inhibitions determined by fluorescence- based method. Results are means from triplicate experiments. [*]The set data are provided in the supporting information for the malaria box.

Table S2. Characterization and IC_{50} values of malaria box compounds number 22 to 41 from plate (A) evaluated for *Babesia divergens* parasites

Compound No.	Compound ID [a]	Set*	IC_{50} (μM) [b]
**C-2**	MMV665785	Probe-like	10.46
**C-3**	MMV665805	Drug-like	4.51
**C-4**	MMV020492	Drug-like	7.60
**C-5**	MMV006937	Drug-like	1.53
**C-6**	MMV666693	Drug-like	2.47
**C-7**	MMV007686	Probe-like	9.94
**C-8**	MMV019066	Drug-like	1.74
**C-9**	MMV000662	Drug-like	3.50
**C-10**	MMV007839	Drug-like	10.19

**C-11**	MMV396680	Probe-like	11.33
**D-2**	MMV665782	Drug-like	3.07
**D-3**	MMV665874	Drug-like	6.35
**D-4**	MMV011567	Drug-like	10.33
**D-5**	MMV006278	Drug-like	3.33
**D-6**	MMV085583	Probe-like	10.11
**D-7**	MMV020885	Probe-like	1.42
**D-8**	MMV006457	Probe-like	2.70
**D-9**	MMV020549	Drug-like	1.67
**D-10**	MMV011259	Drug-like	1.18
**D-11**	MMV396679	Probe-like	1.66

[a] Compounds are designated by their MMV identifier codes; [b] IC_{50} values for each drug were calculated on the fourth day based on the growth inhibitions determined by fluorescence- based method. Results are means from triplicate experiments. [*]The set data are provided in the supporting information for the malaria box.

Table S3. Characterization and IC_{50} values of malaria box compounds number 42 to 61 from plate (A) evaluated for *Babesia divergens* parasites

Compound No.	Compound ID [a]	Set [*]	IC_{50} (μM) [b]
**E-2**	MMV665941	Probe-like	5.29
**E-3**	MMV665878	Drug-like	9.70
**E-4**	MMV000448	Probe-like	10.51
**E-5**	MMV666691	Probe-like	10.55
**E-6**	MMV006455	Drug-like	5.72
**E-7**	MMV666600	Probe-like	10.12
**E-8**	MMV006558	Probe-like	1.72

**E-9**	MMV020548	Drug-like	1.16
**E-10**	MMV011256	Drug-like	1.71
**E-11**	MMV396678	Probe-like	2.62
**F-2**	MMV666021	Probe-like	10.46
**F-3**	MMV665820	Drug-like	4.51
**F-4**	MMV085203	Probe-like	0.83
**F-5**	MMV008956	Drug-like	1.53
**F-6**	MMV007907	Drug-like	2.47
**F-7**	MMV666601	Probe-like	9.94
**F-8**	MMV000570	Probe-like	1.74
**F-9**	MMV001246	Drug-like	3.50
**F-10**	MMV396797	Drug-like	10.21
**F-11**	MMV006087	Drug-like	13.19

[a] Compounds are designated by their MMV identifier codes; [b] IC_{50} values for each drug were calculated on the fourth day based on the growth inhibitions determined by fluorescence- based method. Results are means from triplicate experiments. [*]The set data are provided in the supporting information for the malaria box.

Table S4. Characterization and IC_{50} values of malaria box compounds number 62 to 81 from plate (A) evaluated for *Babesia divergens* parasites

**Compound No.**	**Compound ID** [a]	**Set**[*]	**IC_{50} (μM)** [b]
**G-2**	MMV666062	Probe-like	5.40
**G-3**	MMV665827	Probe-like	2.23
**G-4**	MMV666688	Probe-like	4.86
**G-5**	MMV008416	Probe-like	1.22
**G-6**	MMV006172	Probe-like	1.56
**G-7**	MMV007160	Probe-like	5.65
**G-8**	MMV006427	Drug-like	1.74
**G-9**	MMV019258	Drug-like	10.58

**G-10**	MMV011099	Drug-like	6.67
**G-11**	MMV019110	Drug-like	7.16
**H-2**	MMV665977	Probe-like	1.04
**H-3**	MMV665831	Probe-like	1.61
**H-4**	MMV006203	Probe-like	1.08
**H-5**	MMV020500	Drug-like	1.11
**H-6**	MMV006861	Probe-like	3.45
**H-7**	MMV008294	Probe-like	5.88
**H-8**	MMV020439	Drug-like	1.16
**H-9**	MMV666607	Probe-like	1.03
**H-10**	MMV008138	Drug-like	4.09
**H-11**	MMV396693	Probe-like	0.78

[a] Compounds are designated by their MMV identifier codes; [b] IC_{50} values for each drug were calculated on the fourth day based on the growth inhibitions determined by fluorescence- based method. Results are means from triplicate experiments. [*]The set data are provided in the supporting information for the malaria box.

Table S5. Characterization and IC_{50} values of malaria box compounds number 82 to 101 from plate (B) evaluated for *Babesia divergens* parasites

**Compound No.**	**Compound ID** [a]	**Set***	**IC_{50} (μM)** [b]
**A-2**	MMV665929	Drug-like	0.42
**A-3**	MMV665917	Drug-like	0.28
**A-4**	MMV665979	Drug-like	0.53
**A-5**	MMV666116	Drug-like	0.05
**A-6**	MMV666081	Drug-like	0.06
**A-7**	MMV665906	Drug-like	0.04
**A-8**	MMV665891	Drug-like	0.07
**A-9**	MMV009060	Drug-like	0.05

**A-10**	MMV019313	Drug-like	0.16
**A-11**	MMV000648	Drug-like	0.36
**B-2**	MMV665939	Drug-like	0.25
**B-3**	MMV665789	Drug-like	0.16
**B-4**	MMV665961	Drug-like	0.21
**B-5**	MMV666067	Drug-like	0.05
**B-6**	MMV666093	Drug-like	0.05
**B-7**	MMV665890	Drug-like	0.11
**B-8**	MMV665826	Drug-like	1.05
**B-9**	MMV142383	Drug-like	0.05
**B-10**	MMV007564	Drug-like	0.25
**B-11**	MMV000653	Drug-like	1.43

[a] Compounds are designated by their MMV identifier codes; [b] IC_{50} values for each drug were calculated on the fourth day based on the growth inhibitions determined by fluorescence- based method. Results are means from triplicate experiments. [*]The set data are provided in the supporting information for the malaria box.

Table S6. Characterization and IC_{50} values of malaria box compounds number 102 to 121 from plate (B) evaluated for *Babesia divergens* parasites

Compound No.	Compound ID [a]	Set [*]	IC_{50} (μM) [b]
**C-2**	MMV665935	Drug-like	0.23
**C-3**	MMV665799	Drug-like	0.08
**C-4**	MMV666009	Drug-like	0.14
**C-5**	MMV666071	Drug-like	0.16
**C-6**	MMV666075	Drug-like	0.23
**C-7**	MMV665817	Drug-like	0.24
**C-8**	MMV665902	Drug-like	0.32
**C-9**	MMV006188	Drug-like	0.25

**C-10**	MMV019780	Drug-like	0.33
**C-11**	MMV008149	Drug-like	0.45
**D-2**	MMV665928	Drug-like	0.20
**D-3**	MMV665800	Drug-like	0.07
**D-4**	MMV665953	Drug-like	0.15
**D-5**	MMV666110	Drug-like	0.19
**D-6**	MMV666103	Drug-like	0.18
**D-7**	MMV665899	Drug-like	0.15
**D-8**	MMV665888	Drug-like	0.13
**D-9**	MMV019758	Drug-like	0.05
**D-10**	MMV019746	Drug-like	1.35
**D-11**	MMV019662	Drug-like	1.59

[a] Compounds are designated by their MMV identifier codes; [b] IC_{50} values for each drug were calculated on the fourth day based on the growth inhibitions determined by fluorescence- based method. Results are means from triplicate experiments. [*]The set data are provided in the supporting information for the malaria box.

Table S7. Characterization and IC_{50} values of malaria box compounds number 122 to 141 from plate (B) evaluated for *Babesia divergens* parasites

**Compound No.**	**Compound ID [a]**	**Set[*]**	**IC_{50} (μM) [b]**
**E-2**	MMV665909	Drug-like	0.31
**E-3**	MMV665803	Drug-like	0.27
**E-4**	MMV665946	Drug-like	1.19
**E-5**	MMV665954	Drug-like	0.39
**E-6**	MMV666105	Drug-like	1.33
**E-7**	MMV665857	Drug-like	0.37
**E-8**	MMV007617	Drug-like	1.48

**E-9**	MMV019074	Drug-like	1.20
**E-10**	MMV000788	Drug-like	0.14
**E-11**	MMV001255	Drug-like	0.35
**F-2**	MMV665918	Drug-like	0.36
**F-3**	MMV665796	Drug-like	0.29
**F-4**	MMV666070	Drug-like	0.21
**F-5**	MMV666057	Drug-like	0.16
**F-6**	MMV666108	Drug-like	1.28
**F-7**	MMV665901	Drug-like	0.19
**F-8**	MMV000248	Drug-like	0.26
**F-9**	MMV000498	Drug-like	0.18
**F-10**	MMV000620	Drug-like	0.15
**F-11**	MMV001230	Drug-like	0.62

[a] Compounds are designated by their MMV identifier codes; [b] IC_{50} values for each drug were calculated on the fourth day based on the growth inhibitions determined by fluorescence- based method. Results are means from triplicate experiments. [*]The set data are provided in the supporting information for the malaria box.

Table S8. Characterization and IC_{50} values of malaria box compounds number 142 to 161 from plate (B) evaluated for *Babesia divergens* parasites

Compound No.	Compound ID [a]	Set [*]	IC_{50} (μM) [b]
**G-2**	MMV665914	Drug-like	0.24
**G-3**	MMV665798	Drug-like	0.17
**G-4**	MMV666069	Drug-like	1.17
**G-5**	MMV666072	Drug-like	0.22
**G-6**	MMV666102	Drug-like	0.02
**G-7**	MMV665879	Drug-like	0.001
**G-8**	MMV000563	Drug-like	0.21
**G-9**	MMV019064	Drug-like	1.17

**G-10**	MMV019738	Drug-like	0.13
**G-11**	MMV007571	Drug-like	0.33
**H-2**	MMV665913	Drug-like	0.29
**H-3**	MMV665948	Drug-like	0.15
**H-4**	MMV666061	Drug-like	0.25
**H-5**	MMV666080	Drug-like	0.26
**H-6**	MMV665807	Drug-like	0.01
**H-7**	MMV665850	Drug-like	0.01
**H-8**	MMV665940	Drug-like	0.18
**H-9**	MMV000561	Drug-like	2.18
**H-10**	MMV000634	Drug-like	0.22
**H-11**	MMV011944	Drug-like	0.39

[a] Compounds are designated by their MMV identifier codes; [b] IC_{50} values for each drug were calculated on the fourth day based on the growth inhibitions determined by fluorescence- based method. Results are means from triplicate experiments. [*]The set data are provided in the supporting information for the malaria box.

Table S9. Characterization and IC_{50} values of malaria box compounds number 162 to 181 from plate (C) evaluated for *Babesia divergens* parasites

Compound No.	Compound ID [a]	Set [*]	IC_{50} (μM) [b]
**A-2**	MMV665904	Drug-like	3.78
**A-3**	MMV019762	Drug-like	0.01
**A-4**	MMV019700	Drug-like	2.86
**A-5**	MMV019202	Drug-like	>25
**A-6**	MMV009108	Drug-like	0.12
**A-7**	MMV018984	Drug-like	>25
**A-8**	MMV000972	Drug-like	>25
**A-9**	MMV396719	Drug-like	>25

**A-10**	MMV006820	Drug-like	>25
**A-11**	MMV396705	Drug-like	5.99
**B-2**	MMV665883	Drug-like	4.40
**B-3**	MMV011576	Drug-like	1.74
**B-4**	MMV000760	Drug-like	3.03
**B-5**	MMV020505	Drug-like	0.79
**B-6**	MMV019670	Drug-like	0.59
**B-7**	MMV007808	Drug-like	7.52
**B-8**	MMV306025	Drug-like	>25
**B-9**	MMV396715	Drug-like	0.57
**B-10**	MMV396744	Drug-like	0.55
**B-11**	MMV396704	Drug-like	0.57

[a] Compounds are designated by their MMV identifier codes; [*b*] IC_{50} values for each drug were calculated on the fourth day based on the growth inhibitions determined by fluorescence- based method. Results are means from triplicate experiments. [*]The set are provided in the supporting information for the malaria box.

Table S10. Characterization and IC_{50} values of malaria box compounds number 182 to 201 from plate (C) evaluated for *Babesia divergens* parasites

**Compound No.**	**Compound ID** [a]	**Set** [*]	**IC_{50} (μM)** [*b*]
**C-2**	MMV665806	Drug-like	0.57
**C-3**	MMV000848	Drug-like	2.13
**C-4**	MMV020700	Drug-like	0.95
**C-5**	MMV000911	Drug-like	0.79
**C-6**	MMV020490	Drug-like	1.28
**C-7**	MMV020651	Drug-like	1.76
**C-8**	MMV403679	Drug-like	13.09
**C-9**	MMV396681	Drug-like	0.15

**C-10**	MMV305841	Drug-like	>25
**C-11**	MMV006587	Drug-like	>25
**D-2**	MMV665843	Drug-like	0.38
**D-3**	MMV007881	Drug-like	1.47
**D-4**	MMV020942	Drug-like	1.18
**D-5**	MMV001344	Drug-like	4.69
**D-6**	MMV020275	Drug-like	1.02
**D-7**	MMV007906	Drug-like	2.04
**D-8**	MMV007374	Drug-like	2.63
**D-9**	MMV396595	Drug-like	2.91
**D-10**	MMV396794	Drug-like	0.19
**D-11**	MMV006706	Drug-like	0.03

[a] Compounds are designated by their MMV identifier codes; [b] IC_{50} values for each drug were calculated on the fourth day based on the growth inhibitions determined by fluorescence- based method. Results are means from triplicate experiments. [*]The set are provided in the supporting information for the malaria box.

Table S11. Characterization and IC_{50} values of malaria box compounds number 202 to 221 from plate (C) evaluated for *Babesia divergens* parasites

**Compound No.**	**Compound ID** [a]	**Set** [*]	**IC_{50} (μM)** [b]
**E-2**	MMV665897	Drug-like	1.96
**E-3**	MMV007430	Drug-like	10.24
**E-4**	MMV006767	Drug-like	9.74
**E-5**	MMV001318	Drug-like	1.65
**E-6**	MMV000483	Drug-like	3.49
**E-7**	MMV008212	Drug-like	4.79
**E-8**	MMV008127	Drug-like	6.75
**E-9**	MMV007875	Drug-like	2.00

**E-10**	MMV396632	Drug-like	>25
**E-11**	MMV667487	Drug-like	10.30
**F-2**	MMV000839	Drug-like	1.85
**F-3**	MMV011795	Drug-like	7.04
**F-4**	MMV019918	Drug-like	4.44
**F-5**	MMV008270	Drug-like	2.74
**F-6**	MMV007977	Drug-like	4.03
**F-7**	MMV007978	Drug-like	2.68
**F-8**	MMV056726	Drug-like	1.87
**F-9**	MMV000356	Drug-like	6.23
**F-10**	MMV396633	Drug-like	2.33
**F-11**	MMV000963	Drug-like	2.03

[a] Compounds are designated by their MMV identifier codes; [*b*] IC_{50} values for each drug were calculated on the fourth day based on the growth inhibitions determined by fluorescence- based method. Results are means from triplicate experiments. [*]The set are provided in the supporting information for the malaria box.

Table S12. Characterization and IC_{50} values of malaria box compounds from 222 to 241 from plate (C) evaluated for *Babesia divergens* parasites

**Compound No.**	**Compound ID** [a]	**Set**[*]	**IC_{50} (μM)** [*b*]
**G-2**	MMV019266	Drug-like	0.46
**G-3**	MMV007363	Drug-like	0.46
**G-4**	MMV666599	Drug-like	0.72
**G-5**	MMV019124	Drug-like	0.01
**G-6**	MMV075490	Drug-like	0.10
**G-7**	MMV019127	Drug-like	0.13
**G-8**	MMV396736	Drug-like	0.14
**G-9**	MMV396669	Drug-like	0.11

**G-10**	MMV274073	Drug-like	0.20
**G-11**	MMV006319	Drug-like	0.34
**H-2**	MMV020654	Drug-like	0.50
**H-3**	MMV020660	Drug-like	0.98
**H-4**	MMV001049	Drug-like	0.61
**H-5**	MMV006913	Drug-like	0.001
**H-6**	MMV007791	Drug-like	0.12
**H-7**	MMV000481	Drug-like	0.24
**H-8**	MMV019017	Drug-like	0.22
**H-9**	MMV084940	Drug-like	0.02
**H-10**	MMV396749	Drug-like	0.61
**H-11**	MMV006704	Drug-like	0.63

[a] Compounds are designated by their MMV identifier codes; [b] IC_{50} values for each drug were calculated on the fourth day based on the growth inhibitions determined by fluorescence- based method. Results are means from triplicate experiments. [*]The set data are provided in the supporting information for the malaria box.

Table S13. Characterization and IC_{50} values of malaria box compounds number 242 to 261 from plate (D) evaluated for *Babesia divergens* parasites

Compound No.	Compound ID [a]	Set[*]	IC_{50} (μM) [b]
**A-2**	MMV666025	Probe-like	0.28
**A-3**	MMV666123	Probe-like	7.34
**A-4**	MMV665944	Probe-like	0.69
**A-5**	MMV666026	Probe-like	0.51
**A-6**	MMV666079	Probe-like	2.36
**A-7**	MMV007574	Probe-like	5.81
**A-8**	MMV000304	Probe-like	1.50
**A-9**	MMV019555	Probe-like	0.56

**A-10**	MMV007181	Probe-like	0.52
**A-11**	MMV498479	Probe-like	0.41
**B-2**	MMV006787	Probe-like	0.07
**B-3**	MMV666124	Probe-like	5.88
**B-4**	MMV665994	Probe-like	1.06
**B-5**	MMV666020	Probe-like	2.58
**B-6**	MMV009127	Probe-like	9.98
**B-7**	MMV666597	Probe-like	9.63
**B-8**	MMV007127	Probe-like	4.12
**B-9**	MMV000442	Probe-like	9.69
**B-10**	MMV008474	Probe-like	4.47
**B-11**	MMV007577	Probe-like	0.88

[a] Compounds are designated by their MMV identifier codes; [b] IC_{50} values for each drug were calculated on the fourth day based on the growth inhibitions determined by fluorescence- based method. Results are means from triplicate experiments. [*]The set data are provided in the supporting information for the malaria box.

Table S14. Characterization and IC_{50} values of malaria box compounds number 262 to 281 from plate (D) evaluated for *Babesia divergens* parasites

Compound No.	Compound ID [a]	Set [*]	IC_{50} (μM) [b]
**C-2**	MMV001241	Probe-like	2.47
**C-3**	MMV665908	Probe-like	1.14
**C-4**	MMV665987	Probe-like	0.67
**C-5**	MMV665972	Probe-like	5.59
**C-6**	MMV085471	Probe-like	0.07
**C-7**	MMV000917	Probe-like	2.24
**C-8**	MMV000326	Probe-like	0.51
**C-9**	MMV000443	Probe-like	0.01

**C-10**	MMV019741	Probe-like	0.48
**C-11**	MMV007396	Probe-like	0.40
**D-2**	MMV665934	Probe-like	0.05
**D-3**	MMV665797	Probe-like	0.02
**D-4**	MMV665969	Probe-like	0.02
**D-5**	MMV666109	Probe-like	0.07
**D-6**	MMV019241	Probe-like	0.03
**D-7**	MMV007224	Probe-like	0.03
**D-8**	MMV006753	Probe-like	2.15
**D-9**	MMV000445	Probe-like	0.02
**D-10**	MMV000787	Probe-like	1.55
**D-11**	MMV019995	Probe-like	0.01

[a] Compounds are designated by their MMV identifier codes; [b] IC_{50} values for each drug were calculated on the fourth day based on the growth inhibitions determined by fluorescence- based method. Results are means from triplicate experiments. [*]The set data are provided in the supporting information for the malaria box.

Table S15. Characterization and IC_{50} values of malaria box compounds number 282 to 301 from plate (D) evaluated for *Babesia divergens* parasites

Compound No.	Compound ID [a]	Set[*]	IC_{50} (µM) [b]
**E-2**	MMV665936	Probe-like	0.10
**E-3**	MMV665783	Probe-like	0.33
**E-4**	MMV665949	Probe-like	0.19
**E-5**	MMV665943	Probe-like	0.09
**E-6**	MMV007273	Probe-like	0.07
**E-7**	MMV000699	Probe-like	0.01
**E-8**	MMV000720	Probe-like	0.02
**E-9**	MMV007199	Probe-like	0.15

**E-10**	MMV000604	Probe-like	0.12
**E-11**	MMV008173	Probe-like	0.10
**F-2**	MMV665923	Probe-like	2.68
**F-3**	MMV665786	Probe-like	1.36
**F-4**	MMV666022	Probe-like	0.14
**F-5**	MMV666060	Probe-like	1.92
**F-6**	MMV007228	Probe-like	1.53
**F-7**	MMV666604	Probe-like	0.65
**F-8**	MMV006250	Probe-like	0.17
**F-9**	MMV019690	Probe-like	0.74
**F-10**	MMV000617	Probe-like	0.37
**F-11**	MMV020912	Probe-like	0.05

[a] Compounds are designated by their MMV identifier codes; [*b*] IC_{50} values for each drug were calculated on the fourth day based on the growth inhibitions determined by fluorescence- based method. Results are means from triplicate experiments. [*]The set data are provided in the supporting information for the malaria box.

Table S16. Characterization and IC_{50} values of malaria box compounds number 302 to 321 from plate (D) evaluated for *Babesia divergens* parasites

**Compound No.**	**Compound ID** [a]	**Set**[*]	**IC_{50} (μM)** [*b*]
**G-2**	MMV666125	Probe-like	0.25
**G-3**	MMV665794	Probe-like	0.50
**G-4**	MMV666054	Probe-like	0.001
**G-5**	MMV666095	Probe-like	0.38
**G-6**	MMV006389	Probe-like	0.66
**G-7**	MMV007113	Probe-like	1.52
**G-8**	MMV006882	Probe-like	22.17

**G-9**	MMV007208	Probe-like	0.08
**G-10**	MMV000619	Probe-like	0.03
**G-11**	MMV007557	Probe-like	0.51
**H-2**	MMV665924	Probe-like	16.92
**H-3**	MMV665980	Probe-like	2.63
**H-4**	MMV665971	Probe-like	15.81
**H-5**	MMV666106	Probe-like	0.42
**H-6**	MMV000444	Probe-like	0.47
**H-7**	MMV007275	Probe-like	0.20
**H-8**	MMV006513	Probe-like	0.69
**H-9**	MMV008455	Probe-like	0.52
**H-10**	MMV000621	Probe-like	1.89
**H-11**	MMV006764	Probe-like	1.45

[a] Compounds are designated by their MMV identifier codes; [b] IC_{50} values for each drug were calculated on the fourth day based on the growth inhibitions determined by fluorescence- based method. Results are means from triplicate experiments. [*]The set data are provided in the supporting information for the malaria box.

Table S17. Characterization and IC_{50} values of malaria box compounds number 322 to 341 from plate (E) evaluated for *Babesia divergens* parasites

**Compound No.**	**Compound ID** [a]	**Set**[*]	**IC_{50} (μM)** [b]
**A-2**	MMV665813	Probe-like	3.32
**A-3**	MMV665852	Probe-like	2.11
**A-4**	MMV665810	Probe-like	0.47
**A-5**	MMV007654	Probe-like	9.71
**A-6**	MMV073843	Probe-like	1.50
**A-7**	MMV000340	Probe-like	0.78
**A-8**	MMV396723	Probe-like	1.94
**A-9**	MMV007020	Probe-like	2.23

**A-10**	MMV007041	Probe-like	2.82
**A-11**	MMV638723	Probe-like	15.95
**B-2**	MMV665927	Probe-like	1.66
**B-3**	MMV665898	Probe-like	3.75
**B-4**	MMV665814	Probe-like	1.83
**B-5**	MMV009015	Probe-like	10.28
**B-6**	MMV019881	Probe-like	1.60
**B-7**	MMV086103	Probe-like	10.41
**B-8**	MMV396663	Probe-like	10.46
**B-9**	MMV020243	Probe-like	2.61
**B-10**	MMV667489	Probe-like	10.37
**B-11**	MMV080034	Probe-like	10.97

[a] Compounds are designated by their MMV identifier codes; *b* IC_{50} values for each drug were calculated on the fourth day based on the growth inhibitions determined by fluorescence- based method. Results are means from triplicate experiments. *The set data are provided in the supporting information for the malaria box.

Table S18. Characterization and IC_{50} values of malaria box compounds umber 342 to 361 from plate (E) evaluated for *Babesia divergens* parasites

Compound No.	Compound ID [a]	Set*	IC_{50} (μM) *b*
**C-2**	MMV665894	Probe-like	10.55
**C-3**	MMV665840	Probe-like	10.39
**C-4**	MMV666686	Probe-like	9.86
**C-5**	MMV008160	Probe-like	1.45
**C-6**	MMV009085	Probe-like	1.07
**C-7**	MMV011832	Probe-like	2.73
**C-8**	MMV006303	Probe-like	2.37
**C-9**	MMV396665	Probe-like	10.44

**C-10**	MMV667490	Probe-like	1.96
**C-11**	MMV001041	Probe-like	1.17
**D-2**	MMV665881	Probe-like	0.95
**D-3**	MMV665824	Probe-like	2.58
**D-4**	MMV666687	Probe-like	1.98
**D-5**	MMV008829	Probe-like	10.38
**D-6**	MMV000478	Probe-like	9.903
**D-7**	MMV084434	Probe-like	10.10
**D-8**	MMV396664	Probe-like	10.18
**D-9**	MMV396770	Probe-like	6.00
**D-10**	MMV667491	Probe-like	1.88
**D-11**	MMV667492	Probe-like	1.85

[a] Compounds are designated by their MMV identifier codes; [b] IC_{50} values for each drug were calculated on the fourth day based on the growth inhibitions determined by fluorescence- based method. Results are means from triplicate experiments. [*]The set data are provided in the supporting information for the malaria box.

Table S19. Characterization and IC_{50} values of malaria box compounds number 362 to 381 from plate (E) evaluated for *Babesia divergens* parasites

**Compound No.**	**Compound ID** [a]	**Set**[*]	**IC_{50} (μM)** [b]
**E-2**	MMV665836	Probe-like	2.16
**E-3**	MMV665830	Probe-like	2.02
**E-4**	MMV007474	Probe-like	2.27
**E-5**	MMV000753	Probe-like	3.49
**E-6**	MMV007591	Probe-like	2.52
**E-7**	MMV020750	Probe-like	11.71
**E-8**	MMV000704	Probe-like	2.15
**E-9**	MMV006169	Probe-like	2.06

**E-10**	MMV396717	Probe-like	12.08
**E-11**	MMV645672	Probe-like	10.69
**F-2**	MMV665882	Probe-like	5.12
**F-3**	MMV665809	Probe-like	6.30
**F-4**	MMV011895	Probe-like	9.947
**F-5**	MMV020403	Probe-like	6.45
**F-6**	MMV666692	Probe-like	12.25
**F-7**	MMV006962	Probe-like	10.68
**F-8**	MMV396594	Probe-like	10.45
**F-9**	MMV396726	Probe-like	4.71
**F-10**	MMV396652	Probe-like	10.20
**F-11**	MMV006522	Probe-like	3.22

[a] Compounds are designated by their MMV identifier codes; [b] IC_{50} values for each drug were calculated on the fourth day based on the growth inhibitions determined by fluorescence- based method. Results are means from triplicate experiments. [*]The set data are provided in the supporting information for the malaria box.

Table S20. Characterization and IC_{50} values of malaria box compounds number 382 to 401 from plate (E) and diminazene aceturate evaluated for *Babesia divergens* parasites

**Compound No.**	**Compound ID** [a]	**Set**[*]	**IC_{50} (µM)** [b]
**G-2**	MMV665812	Probe-like	3.91
**G-3**	MMV665864	Probe-like	0.57
**G-4**	MMV011436	Probe-like	2.23
**G-5**	MMV128432	Probe-like	10.19
**G-6**	MMV007092	Probe-like	1.94
**G-7**	MMV001239	Probe-like	10.32
**G-8**	MMV667486	Probe-like	2.56

**G-9**	MMV000986	Probe-like	2.55
**G-10**	MMV011522	Probe-like	2.93
**G-11**	MMV396635	Probe-like	1.95
**H-2**	MMV665875	Probe-like	2.29
**H-3**	MMV665886	Probe-like	3.25
**H-4**	MMV011438	Probe-like	10.06
**H-5**	MMV666689	Probe-like	4.65
**H-6**	MMV007764	Probe-like	2.30
**H-7**	MMV006656	Probe-like	10.27
**H-8**	MMV007285	Probe-like	3.39
**H-9**	MMV667488	Probe-like	2.85
**H-10**	MMV019199	Probe-like	10.26
**H-11**	MMV006825	Probe-like	4.19
**-**	DA	Drug-like	0.13

[a] Compounds are designated by their MMV identifier codes; [b] IC_{50} values for each drug were calculated on the fourth day based on the growth inhibitions determined by fluorescence- based method. Results are means from triplicate experiments. [*]The set data are provided in the supporting information for the malaria box. DA, diminazene aceturate (positive control drug)

Table S21. Viability test results of potent MMV hits evaluated for *Babesia divergens*

**Compound ID** [a]	**Drug concentrations (μM)** [b]			
	**25**	**10**	**1**	**0.1**
MMV665807	-	-	-	-
MMV665850	-	-	-	-
MMV019124	-	-	-	-
MMV006913	-	-	-	-
MMV019995	-	-	-	-
MMV000699	-	-	-	-
MMV666054	-	-	-	-

[a] Compounds are designated by their MMV identifier codes. [b]any value calculation was dependent on the fluorescence assay in three different treatment of experiments. Every concentration was done in triplicate in each experiment. + = viable; − = dead
